# Differential DNA methylation associated with anti-dsDNA autoantibody production in systemic lupus erythematosus

**DOI:** 10.1186/ar3940

**Published:** 2012-09-27

**Authors:** SA Chung, J French, KE Taylor, E Elboudwarej, HL Quach, LF Barcellos, LA Criswell

**Affiliations:** 1 Rosalind Russell Medical Research Center for Arthritis, University of California, San Francisco, CA, USA; 2 School of Public Health, University of California, Berkeley, CA, USA

## Background

Aberrant DNA methylation has been implicated in the pathogenesis of systemic lupus erythematosus (SLE), with less DNA methylation observed in SLE patients compared with healthy controls. We sought to determine whether DNA methylation differences are also associated with anti-dsDNA autoantibody production in SLE.

## Methods

Genomic DNA from peripheral blood leukocytes was isolated from 326 SLE patients, including 158 anti-dsDNA-positive and 168 anti-dsDNA-negative. All SLE cases were female, of European descent, and had never smoked. DNA methylation profiles were initially characterized on a subset of patients (*n *= 208, with dsDNA-positive cases matched to dsDNA-negative cases on age and disease duration) using the Illumina HumanMethylation27 Beadchip (27 k chip). Subsequently, all patients (*n *= 326) were characterized for the HumanMethylation450 array, and analyses focused on the most strongly associated regions from the 27 k methylation data. Paired *t *tests were used to identify site-specific methylation differences associated with anti-dsDNA autoantibody production in the initial phase, with *P *< 1.8 × 10^-6 ^(Bonferroni corrected) considered statistically significant. Genome regions that demonstrated statistically significant associations with dsDNA status were further assessed using the 450 k methylation data.

## Results

Overall, less methylation was observed in anti-dsDNA positive patients compared with dsDNA-negative patients. Analysis of the 27 k methylation data demonstrated decreased methylation of a CpG site near *SOCS2 *(*P *= 3.5 × 10^-7^), an inhibitor of the STAT family of transcription factors, as well as a site near *GGT1 *(*P *= 4.3 × 10^-3^). In addition, increased methylation of two CpG sites in *PRIC285*, a transcriptional co-activator for nuclear receptors, was significantly associated with anti-dsDNA autoantibody production (*P *= 1.9 × 10^-7 ^and 3.4 × 10^-7^). Subsequent analysis of 114 methylation sites from the 450 k methylation data in these regions demonstrated very strong association with the methylation site cg22764925, located in the 5'-UTR region of *GGT1 *(*P *= 2.8 × 10^-11^), with an average difference of 7.7% between methylation levels of anti-dsDNA-positive and anti-dsDNA-negative patients. The methylation site cg11738543, and several other CpG sites in the *SOCS2 *gene, were also associated with anti-dsDNA antibody status (*P *= 4.2 × 10^-8^).

## Conclusion

These findings suggest that abnormal methylation patterns of specific genes, including *GGT1 *and *SOCS2*, may contribute to autoantibody production in SLE. Studies of DNA methylation and other epigenetic modifications may help elucidate biologic mechanisms involved in the pathogenesis of SLE.

